# Moving from silos to synergies: strengthening governance of food marketing policy in Thailand

**DOI:** 10.1186/s12992-022-00825-5

**Published:** 2022-03-09

**Authors:** Sirinya Phulkerd, Yandisa Ngqangashe, Jeff Collin, Anne-Marie Thow, Ashley Schram, Carmen Huckel Schneider, Sharon Friel

**Affiliations:** 1 Institute for Population and Social Research, Mahidol University, 999 Phutthamonthon 4 Road, Phutthamonthon, Nakhon Pathom, 73170 Thailand; 2 School of Regulation and Global Governance, Australian National University, Canberra, Australia; 3 Global Health Policy Unit, School of Social & Political Science, University of Edinburgh, Edinburgh, UK; 4 Menzies Centre for Health Policy and Economics, School of Public Health, University of Sydney, Sydney, Australia

**Keywords:** Food marketing, Institutional process, Governance, Non-communicable diseases, Nutrition, Ultra-processed foods

## Abstract

**Background:**

Governance processes play an important role in shaping the formulation and implementation of policy measures such as restrictions on marketing of ultra-processed foods. However, there is limited analysis of the factors that affect governance for nutrition, especially in low- and middle-income countries such as Thailand and the Southeast Asia region. This study aimed to examine governance factors that create opportunities and challenges for the introduction of policy to restrict food marketing in Thailand, in line with the WHO recommendations to restrict food marketing to children.

**Methods:**

A qualitative study design was used. Interviews were conducted with 20 actors with experience and in depth knowledge of food marketing in Thailand, including government, civil society, industry and international organisations. Open questions were asked about experiences and perceptions of the governance processes related to policies for restricting food marketing in Thailand. Themes were derived from the 3-i Framework which relates to interests, ideas and institutions influencing the introduction of food marketing policy were identified and analysed using abductive methods.

**Results:**

Actors viewed institutional challenges as a significant barrier to advancing effective regulation of food marketing. Three major clusters emerged from the data: interests (priorities, relationships), institutions (formal structures, informal structures, broader institutional strategies), and ideas (norms). The study has three major findings in relation to these factors, highlighting the influence of formal structures, institutional interests in food marketing issues, and ideas in promoting multisectoralism. The siloed nature of policymaking was reflected in the government failing to stimulate engagement among key actors, posing challenges for implementation of effective policy change. Contested interests led to disagreements between actors over food marketing agenda and thus competing policy priorities. Consistent with these findings, the lack of effective mechanisms to promote multisectoral coordination across diverse actors reinforced barriers to policy change.

**Conclusion:**

The findings highlight ongoing challenges to the government’s aim to strengthen policy to restrict food marketing which, without greater coordination in governance mechanisms, will hinder effective regulation and the achievement of public health goals. This analysis suggests that the Government should prioritise the development of a holistic, multisectoral approach to improve governance for better nutrition outcomes by overcoming policy silos.

## Background

Food marketing is an important environmental factor that influences children’s and adult’s knowledge, preferences and consumption of foodstuffs related to non-communicable diseases (NCDs) [1]. Internationally, ultra-processed foods (UPF) such as sugar-sweetened beverages, confectionery and savoury snacks are the most frequently marketed unhealthy products, especially to children on television [1]. The World Health Organization (WHO) developed a set of recommendations on the marketing of foods and non-alcoholic beverages to children, which will contribute to reducing the impact of UPF marketing [2]. Reducing exposure to UPF marketing has emerged as a critical factor in helping to lower the risk of diet-related NCDs.

Sales growth of UPF is most pronounced in lower-middle income and upper-middle-income countries, particularly in South and Southeast Asia [3], and is expected to lead to consumption levels commensurate with high-income countries by 2035 [4]. In Thailand, UPF products such as instant noodles, sugar-sweetened beverages, salty snacks, bakery products, and processed meat products are increasingly consumed by Thai people across all age groups [5].

However, the Thai government has yet to implement substantive policy action. In 2008 Thailand launched an initiative to restrict advertising of unhealthy food and beverage products including UPF on radio and television. However, this effort stalled following a change in government, with responsibility for the policy transferred from the Government Public Relations Department to Office of The National Broadcasting and Telecommunications Commission [6]. Despite an ongoing effort to revive this restriction by developing a national food marketing restriction guideline led by Department of Health, significant challenges remain to its adoption and implementation. Governance processes that enable the necessary intersectoral “joint action” between government departments, civil society, technical experts and the private sector towards improved nutrition of Thai people need to be examined.

Governance plays an important role in shaping the formulation and implementation of policy measures [7]. Governance can be defined as “the exercise of economic, political and administrative authority to manage a nation’s affairs [8].” Therefore, the quality and efficiency of government processes can crucially affect the development and implementation of public policies.

The introduction of effective policy actions can be impeded by various factors relating to government processes, such as a lack of leadership, poor strategic capacity and limited authority of designated lead institutions, alongside power asymmetries and divergent interests among actors in the policy process including government, international organisations, civil society and industry actors [9–11]. Moreover, if government actors do not have incentives to cooperate with one another, siloed and fragmented policy processes can hinder policy formulation and implementation [12].

There is limited analysis of these governance issues in the field of nutrition [9, 13], especially in low- and middle-income countries such as Thailand and the Southeast Asia region. This study aimed to examine the governance processes that create opportunities and challenges for the development of policy to restrict food marketing in Thailand. The findings from this study will provide valuable lessons for other middle-income countries looking to implement policy to restrict food marketing.

## Methods

### Study design and participants

This study used an exploratory qualitative design. Semi-structured interviews were conducted with Thai actors related to food marketing, including government, civil society, research and academic, industry, and international organisations. A list of relevant actors was initially drawn from secondary data sources, including governmental and non-governmental websites and documents, and Internet searches. Purposive sampling strategy was used and then supplemented with snowballing from existing interviews. Twenty-nine actors were invited to interview, and 20 agreed to participate (Table 1).

**Table 1:** Description of the sampled interviewees

Group and subgroup	Respondents
Government (GO)	
- Health	2
- Food and nutrition	2
- Consumer protection	1
- Education	1
- Media	1
Civil society (CV)	
- Health	2
- Food and nutrition	1
- Consumer protection	1
- Media	1
Technical expert (TE)	
- Food and nutrition	3
- Food marketing	1
- Media	1
Industry sector (IS)	2
- Food industry	1
- Advertisement industry	1
International organisation (IO)	1
**Total**	**20**

### Data collection

The interviews were conducted from May to September 2020, by SP. As the research was exploratory, open questions were asked in order to capture each person/organisation’s experience and perception of institutional processes that create opportunities and challenges for policy creation related to the introduction of policy to restrict food marketing in Thailand. Guiding questions were set by SP and YN based on the 3-i framework with three mutually constituted features: interests, institutions and ideas (detailed description of the framework is provided in data analysis section).

Each interviewee was informed as to the purpose of the research and given an information sheet before giving signed consent to participate in the interview study. Each interview was digitally recorded and all interviewees consented to audio recording. Verbatim transcription was prepared by SP. Every effort has been made to anonymise the individual participants, reflecting the politically sensitivities attached to these issues. Attribution is therefore made by sector of organisation and participant number only. The interviews lasted between 1 h and 1.5 h.

### Data analysis

For interviews conducted in Thai, the transcripts were translated to English by a native-speaking professional translator, and crosschecked by SP. Once all the interviews were conducted, the coding was done by SP and analysis of the emerging themes was done by the whole research team in an iterative process. The coded categories were derived from the data and a pre-existing theoretical framework which is 3-i Framework (described below). This is an iterative process of abstraction where units of the data (words and sentences from the interview transcripts) relating to the topic of instrumental processes were identified, combined and grouped with similar content to form major themes and subthemes. Coder reliability checking process was carried out with three independent coders (SP, JC and YN) to analyse and compare the results, and discuss if there were any discrepancies. The analysis was performed in NVivo 12 software by SP. The analytical framework that were explored throughout the interviews were informed by the 3-i Framework, as outlined in Table 2. This political science framework poses that policy development is influenced by three mutually constituted features: interests (i.e., actor agendas and their relative power and influence), institutions (i.e., the rules and structures in place) and ideas (i.e., the knowledge and discourses drawn on and the values they reflect) [14–17]. Interests, ideas and institutions are known to influence the development and implementation of public policy [14, 18]. However, little is empirical evidence exists of the influence of interests, ideas and institutions in the Thai nutrition policy context. The 3Is framework enables a guided exploration of each individually and also their interaction, and how they shape policy developments in public health related areas. The framework has been utilized in studies of Irish social security policy changes [19–21], politics of national HIV policy [22], and challenges of food systems research [23]. Therefore, this framework is considered an appropriate analytical framework for this study’s aim.

**Table 2:** Concepts explored in the interviews

3-i Framework	Concepts	Example questions
Interests	Priorities, relationships	What are actors’ priorities? Why are these important? Who do the actors work with? What drives these collaborations and where do they take place? What should be considered when making the policies (i.e., actions adopted or proposed by governments) to support healthy diets?
Institutions	Formal/informal structures	Which formal or informal processes are used by actors? How do these advance / undermine policy?
	Broader institutional strategies	What are the policies, strategies or frameworks that actors use to influence policy development?
Ideas	Norms, multisectoralism, conceptualizations of the policy problem and marketing restrictions	How do norms, discourses and practices shape policy?

## Results

### Interests

Several actors from health sector identified restricting unhealthy food marketing such as UPF as an issue that the government should be prioritizing. Civil society actors and technical experts remarked on the importance of a clear understanding of food marketing practices, especially their concerns on the adequacy of existing controls given developments in digital media, but not specific to marketing of food products.

*Traditional forms of controlling the marketing of consumer products is not keeping pace with the advertisers and marketing agencies* (TE1).

A number of actors from government, technical expert and international organisation supported tighter controls on food marketing and some viewed UPF as priority target or “*low-hanging food to target”* (IO1). One government actor supported that “*if I had to choose just one target, it would be the UPF that has to be taken out of the Thai diet”* (GO3). Meanwhile, another government official indicated that “*in any policy discussion, the health of the consumer is always the sub-text. ‘Safety first’ is the overriding principle”* (GO5).

Participants from government, technical experts and food and advertisement industry acknowledged the existence of collaboration within and between a range of government and non-governmental organisations. The connection between government organisations and the food industry was frequently noted, with some participants linking such relationships to the reluctance among government actors to intervene and/or the desire to align with interests of key food industry actors.

*It would not be a constructive approach since industry is already skeptical of us. They do not want any interference in their business. […] if we classify foods as “healthy” or “unhealthy” then it is a form of stigmatizing products or parts of the food industry, and that will create enemies in a hurry. If we portray a product as evil, then we will never be able to get cooperation from Industry to improve food marketing policy and practices* (TE1).

Some government, civil society and international actors cited tensions between institutional interests across economic and health spheres. Economic development was prioritised over health by some government actors in non-health departments, therefore obstructing a food marketing regulation agenda. One civil society actor expressed difficulty to work with some Ministries due to conflicting economic interests which make food marketing issues highly salient.

*My view is that it would be more difficult to try to push change through the Ministry of Commerce since they are so closely linked with the economy. Thus, they don’t really want to team up with us (since our campaign would reduce sales of certain products if successful)* (CV4).

### Institutions

#### Formal structures

Participants from all sectors except the food and advertisement industries described inadequacies of existing organisational structures within government and coordinating mechanisms as posing challenges to introducing policy restrictions on food marketing. This was attributed to national institutional arrangements that had been insufficient to support and link relevant actors to help introduce such policies, especially regarding authority, cross-sectoral issues, and power imbalances.

*So the center of power depends on which aspect of marketing you are looking at. […], there are two power centers: The Office of the Consumer Protection Board and the FDA [Food and Drug Administration]. If they can join forces and draft new laws, and then work with the Ministry of Digital Economy and Society and the NBTC [Office of the National Broadcasting and Telecommunications Commission] to develop some controls, then that should provide broad protections* (TE1).

Relevant government departments and agencies were regarded as having fragmented responsibilities and restrictions on their roles, and as such there is reluctance among the departments to extend their mandates or take responsibility for action in this space. It was perceived by one international actor (IO1) that food marketing agenda is *“so cross cutting”* as it “*sits in many different agencies, so it has many masters and no real owners.”*

Government departments were viewed as working separately from each other due to different policy objectives, limited authorities and “*the changing powers-that-be”* (CV4). Accordingly, they failed to find ways of working across silos, and as such failed to effect policy.

*the NBTC had control over the dozens of TV channels on air, but they did not want to exert that control. There was no agency to step up to control content. The Board of the NBTC was basically a policy unit, while the units with the mandate to act were the line agencies within the NBTC. But those agencies first needed a policy from the Board in order to act. Further, the agencies didn’t feel they had enough manpower to implement that kind of policy on broadcast content. So, they focused on other areas that were easier to control* (CV4).

To tackle the problem of siloed working, one government actor reported that their department has created formal mechanisms to “*improve collaboration through the task force mechanis”* so that it can *“advocate on this very [marketing] issue and produce some concrete results”* (GO3). Other government actors described a primarily “rule-bound sense” of policy process with limited discussion on formal mechanisms or dynamics.

*we would like to be involved more with online marketing. Now, all we can do is to ask for the cooperation of the online platforms to police themselves and alert us if something does not meet [our] standards or is unsafe. We have some MOUs in this regard […] We are restricted in how far we can reach into marketing practices. So, there will have to be changes to the law. Somebody has to set some standards. If we go too far in controls, we could be sued* (GO5).

However, these formal mechanisms were viewed by technical experts and civil society actors as insufficient for promoting coordination across departments and sectors and advocating for policy and better nutrition. One technical expert felt that “*the legal process is too slow and inflexible to adapt to the rapidly changing marketplace. […] It’s like we are chasing our own shadow”* (TE1).

#### Informal structures

Some participants viewed informal structures, spaces or networks such as personal contacts and informal one-to-one meetings with policy makers or law makers themselves, as creating significant opportunities for influencing policy decisions. Some government actors reported setting up informal meetings at first to “*set some targets and define the stakeholders”* (GO3) and “*check existing evidence and explore who might support or oppose the policy”* (GO7). Civil society and technical actors used informal channels to access information from other actors in policy network.

*I think it would be more in the non-formal meetings where the final decision takes place. Usually, after the large, open meetings take place, then there will be a smaller group which meets to take stock of what the direction should be. The policy makers have their own, larger agendas which they need to adhere to. Sometimes, they realize that it won’t be possible to get a consensus among the academic/technical specialists since there is not enough evidence. So, they proceed with an ad hoc decision based on an informal consultation* (TE4).

#### Broader policy context

The WHO’s “Set of recommendations on the marketing of foods and non-alcoholic beverages to children” [2] was identified by several interviewees as a national and/or regional roadmap that could guide the government on designing new policies to reduce the impact on marketing of unhealthy foods to children.

*Currently, there is more collaboration among ASEAN member countries, and some have stepped up to host the advocacy effort to control cross-border marketing. A minimum set of recommendations for advocacy is included in the 2025 Plan of ASEAN, and members will review the full set of WHO recommendations to see which they can collaborate on* (GO3).

Some actors suggested a more comprehensive, multi-sectoral approach for food marketing that can empower people, families and communities to take control of their media use behaviours.

*It’s not like there is one organization you can appeal to for change [in food marketing]. It has to be a family-driven and community-based approach to control online media. What is more, people on the Internet are using Avatars to hide their identity. So, it is becoming increasingly difficult to identify who is who on the Internet. So, this problem extends way beyond food marketing. It has to be addressed holistically – not just sector by sector. This is because it is threatening to transform entire societies and economies. So, any approach has to be broader than health* (TE4).

Some government and civil society actors also remarked on “catalytic events” that pose opportunities for increasing marketing restrictions by creating venues for policy discussions where nutrition actors can seek a seat at the table and position nutrition within the larger policy issues such as discussions of *“Thailand 20-year Strategy”, “Sustainable Development Goals”* and “*Global NCD targets,”* and *“visits by United Nations Interagency Task Force on NCDs”* (GO2, GO7, CV2).

It was noted by one food industry actor that food and nutrition standards setting should be primarily based on societal conditions such as modernisation and food innovation.

*With modernization, people now can buy processed food and beverage that can be stored almost indefinitely and still be pure and safe to consume when needed. This also means that nutritious food can be distributed to even the most remote parts of the world and still retain its flavor and nutritional value. There is also the convenience factor of being able to buy a food or beverage and then consume it at one’s leisure. As society becomes more mobile, processed food and drink will become indispensable. We will never go back to ancient times when all food and water had to be collected and consumed where one lived* (IS1).

### Ideas

Participants from all sectors discussed the influence of “technical norms” on government decision making, through appeals for evidence-based decision-making, or by “external reference points” to the evidence produced by the WHO or other authoritative sources regarding efficacy.

*We have had repeated consultations with the WHO in this area about recommendations for control of food marketing […] If the scientific evidence is strong, then it is easier to forge cross-sector collaboration […] then industry will cooperate* (GO3).

Some government in health sector and civil society actors remarked on the idea of “social proofing” where they use experiences of other governments where their adopted policy is successful to help them determine actions.

*Japan had been successful in controlling advertising by working through their Ministry of Commerce. They used a strategy of equality in advertising. […] the Ministry of Commerce argued successfully that the producers had created a virtual monopoly by using prize drawings as an incentive. […] suggested that we try to work through Thailand’s Ministry of Commerce* (CV4).

Despite these acknowledgements, some government actors felt about unclear evidence, proving potential harm of unhealthy food marketing to health.

*I don’t think that connection is that clear yet. Marketing is geared toward generating profits and expanding the business. Health is probably not seen as an additive factor in that equation. Health is a rather complex and abstract concept, and food is just one component of that. […] It is too complex to say that this food will always be good for you or that another food is always harmful* (GO5).

The commitment to evidence-based policy making was also articulated by one food industry actor. This was accompanied by raising concerns about the relevance and quality of research findings and bias on selection of data for analysis, drawing on examples relating to sugar-sweetened beverage taxation.

*I respect any scientific evidence if collected in good faith. But some of the food quality studies might have suffered from methodological weaknesses. They might not have done control trials. There is the case of the “Australian paradox” which found that after implementing controls on sugar consumption, the rates of diabetes and related NCD did not decline. New Zealand has looked at the impacts as well, and they decided not to impose a sugar tax since there was no clear evidence that doing so would improve health outcomes and reduce NCD related to sugar consumption. So, I think Thailand’s imposing a sugar tax was probably not too fair. However, if industry funds the research, then people won’t believe the findings* (IS1).

There was a strong emphasis on consensus building with food industry as important influence on agenda-setting in policy to restrict food marketing.

*instead of trying to punish industry and food marketing of unhealthy foods, we can take a more positive approach and encourage them to focus more on these food innovations that are becoming popular around the world. That way, we would be allies and not adversaries* (CV4).

## Discussion

This study constitutes a first attempt at understanding governance processes and factors that have influenced government policy development related to the restriction of food marketing in Thailand. The study examined three clusters of factors that affect this policy introduction: interests (priorities and relationships), institutions (formal structures, informal structures, broader institutional strategies), and ideas (norms, multisectoralism, conceptualizations of policy problem and marketing restriction). Three linked major findings arose from the analysis: silos in government; contested institutional interests over food marketing agenda; and a lack of a holistic, multisectoral approach.

The most notable finding here relates to institutional factors - silos in government. This factor was raised repeatedly among the participants as having a major influence on the introduction of policy to restrict food marketing in Thailand. As regulating food marketing, including UPF, requires inter-sectoral actions involving a range of health and non-health actors, a failure to create opportunities for engagement among these actors impedes policy development. This reflects findings in other countries. For example, in Fiji, a lack of clear institutional responsibility for marketing restrictions (in particular, between health and economic sectors) was identified as a policy barrier to restricting food marketing to children [9]. Silos are often understood in the literature as barriers to communication flow and information exchange [24]. As such, they pose threats to decision-making, use of resources, and service delivery, as well as resolving cross-cutting problems and collaboration with different actors. Despite calls for breaking down silos [25, 26], like many countries such as Fiji, the problem of silos continues to characterize policymaking in Thailand, and radical reform will be politically and administratively difficult in a unitary country with a highly centralised, hierarchical administrative system composed of large government departments [27]. The present study suggests that the first step is to shift towards what have been referred to as “dancing silos” that are “*more flexible, permeable, interactive and transparent, while keeping their typical strengths and their specific functions in different administrative cultures* [28].” This can be facilitated via informal coordinating platforms or networks alongside monitoring of political directives and actors’ activities. This should be paired with support for capacity-building and skills development in the public service to overcome silo-thinking and planning.

The next key finding relates to contested institutional interests around food marketing. These points of contention occurred primarily among government actors and the food and advertisement industry actors. While technical experts supported control of unhealthy food marketing, some government actors addressed food safety and the food industry prioritised a consumer-demand driven approach. These differences may be the product of structural factors such as silos, diversity in institutional perspectives and interests that shape behaviors and power relations among actors involved in the policy process, and thus competing policy priorities. Previous evidence confirms that structural factors can impede progress in forming and implementing a coherent nutrition related agenda [29–31]. However, these factors could be molded or aligned through strengthening core policy communities. For example, civil society mobilisation can enhance the saliency of food marketing issues on the political agenda, such as promoting or facilitating a larger policy discourse within which nutrition can be strategically framed through national and global targets. Similarly, technical experts can help frame food marketing issues in relation to other challenges such as poverty, as well as other social and economic priorities.

The third key finding is a consequence of the two preceding findings, evident in the lack of a holistic, multi-sectoral approach for food marketing. Concerns about the fragmented responsibility for food marketing across different ministries caused by incoherence and lack of coordination between actors were raised in this study. The participants indicated a need for a holistic, multi-sectoral approach. By addressing the determinants of health such as UPF consumption, multisectoral action and its coordination are needed to promote these health-enhancing actions from non-health sectors. This approach is clearly emphasized in WHO’s framework for implementing the set of recommendations on the marketing of foods and non-alcoholic beverages to children for successful implementation [32]. However, this has not been put into actions in Thailand. This approach is critical for not only the achievement of unhealthy food marketing control, but also the global targets on NCDs and nutrition and the Sustainable Development Goals by 2030. Therefore, systematic multidisciplinary planning [33] is needed to treat the food marketing issues holistically and produce a combination of policy or project initiatives in several development sectors. This should include raising awareness of the multisectoral nature of food marketing among actors as well as creating their vision for working multisectorally and managing it innovatively, with more enabling institutional environment in terms of human, financial or technical resources.

This study set out to identify the interests, ideas and institutional-related governance factors that have influenced the introduction of policy to restrict food marketing in Thailand in recent decades. In doing so, we also endeavour to draw lessons from the analysis to inform the introduction and implementation of the global targets on NCDs and nutrition and the Sustainable Development Goals. The three linked major findings of this study have implications for middle-income countries that have silos-dominant administrative systems, and which often face the same challenges in coordinating government departments for advancing nutrition outcomes through policies to restrict food marketing. The findings also suggest policy learning as an important process in which information and experiences can be useful for guiding the design and implementation of public policies. Other countries can provide opportunities from learning from experiences of others, or the past, in policy-making processes for better policies developed in the future.

This study has some limitations. A range of actors from different sectors were recruited to the study, but there were less representatives from the food and advertisement industries than the other types. Therefore, perspectives of this sector may not be fully reflected. Despite this limitation, the influence of the industry actors included in the analysis reflects what has been identified in previous studies. The food marketing and governance issues identified by the interviewees, and examples given, may pre-date the current food marketing movement. However, as the interviewees also identified current and ongoing efforts to address the challenges, the governance issues were considered outstanding at the point of the interview. Linguistic challenges are also acknowledged. This study was cross-language (Thai and English) qualitative research and as such a language barrier was present between researchers and participants especially through the use of a translator. However, the researchers evaluated the work of translators by conducting backward translation for the first few translated transcripts before analysing data, to minimise the influence of linguistic differences.

## Conclusions

This exploratory study captured a diverse range of actor perspectives on the development and implementation of policy to restrict food marketing, through the lens of governance in Thailand. Specifically the analysis provides an understanding of the interests, ideas and institutional challenges and opportunities to strengthening the introduction of policy to restrict food marketing in middle-income countries in this critical decade running up to achieving global health targets in 2030.

The findings suggest that the Government of Thailand should make efforts to improve governance for better nutrition outcomes in key following areas: overcoming policy silos; seeking agreements over food marketing agenda; and using a holistic, multisectoral approach. The findings highlight ongoing challenges to the government’s aim to strengthen the introduction of policy to restrict food marketing, without greater coherence and coordination in governance mechanisms, will prevent achievement of policy to restrict food marketing and reduce exposure to UPFs.

## Data Availability

The datasets generated and/or analyzed during the current study are not publicly available to ensure privacy of interviewees. Access to anonymized interview transcripts and archival data is available from the corresponding author on reasonable request.

## Abbreviations

CV: Civil society
FDA: Food and Drug Administration
GO: Government
IO: International organization
IS: Industry sector
NBTC: Office of the National Broadcasting and Telecommunications Commission
NCDs: Non-communicable diseases
TE: Technical expert
UPF: Ultra-processed foods
WHO: World Health Organization
